# The tellurite resistance gene cluster of pathogenic bacteria and its effect on oxidative stress response

**DOI:** 10.1007/s12223-024-01133-8

**Published:** 2024-01-23

**Authors:** Silvia Vávrová, Jozef Grones, Katarína Šoltys, Peter Celec, Ján Turňa

**Affiliations:** 1 Faculty of Natural Sciences, Department of Molecular Biology, Comenius University in Bratislava, Bratislava, Slovak Republic; 2 Faculty of Natural Sciences, Department of Microbiology and Virology, Comenius University in Bratislava, Bratislava, Slovak Republic; 3 Faculty of Medicine, Institute of Molecular Biomedicine, Comenius University in Bratislava, Bratislava, Slovak Republic; 4 Faculty of Medicine, Institute of Pathophysiology, Comenius University in Bratislava, Bratislava, Slovak Republic

**Keywords:** Oxidative stress response, Pathogenesis, Pathogen evolution, Tellurite resistance gene cluster, Uropathogenic *Escherichia coli*

## Abstract

Tellurite resistance gene clusters have been identified in numerous pathogenic bacteria, including clinical isolates of *Escherichia coli*. The rareness of tellurium in host organisms and the noncontaminated environment raises a question about the true functionality of tellurite resistance gene clusters in pathogenesis and their possible contribution to bacterial fitness. The study aims to point out the beneficial effects of the tellurite resistance gene cluster of pathogenic bacteria to survive in ROS-rich environments. Here, we analysed the bacterial response to oxidative stress conditions with and without tellurite resistance gene clusters, which are composed of *terWY1XY2Y3* and *terZABCDEF* genes. By measuring the levels of protein carbonylation, lipid peroxidation, and expression changes of oxidative stress genes upon oxidative stress, we propose a tellurite resistance gene cluster contribution to the elimination of oxidative damage, potentially increasing fitness and resistance to reactive oxygen species during macrophage attack. We have shown a different beneficial effect of various truncated versions of the tellurite resistance gene cluster on cell survival. The *terBCDEF* genes increased the survival of *E. coli* strain MC4100 by 13.21%, *terW* and *terZABCDEF* by 10.09%, and *terWY1XY2Y3* and *terZABCDEF* by 25.57%, respectively. The ability to survive tellurite treatment is the most significant at 44.8% in wild clinical strain KL53 compared to laboratory strain *E. coli* MC4100 due to a complete wild-type plasmid presence.

## Introduction

Tellurium oxyanions in general are toxic for most organisms. Tellurium is a rare element of the Earth’s crust with a natural abundance of only about 10^−2^ to 10^−8^ ppm (Belzile and Chen 2015). The lack of available analytical methods is the limiting factor for more accurate and more recent values of tellurium compounds in water, soil, and atmosphere. Seawater values are of the order of tenths to tens of nanograms per litre, with 2–3 orders of magnitude difference. Coal combustion was described as a main anthropogenic source of atmospheric tellurium and volcanism and biomethylation as natural atmospheric sources of tellurium. The value 0.006 mg/kg is the world’s average soil tellurium content (Filella et al. 2019). The scarcity of tellurium compounds in the environment suggests that bacteria seemingly have no reason to evolve resistance against them. Only a handful of microorganisms that inhabit extreme ecological niches with a higher concentration of tellurium compounds are naturally resistant. A few mining environments were described as tellurite-rich environments, even though tellurium was not the target element for mining. Discarded residues, however, had relatively higher concentrations of tellurium-containing species (Farias et al. 2022). The increasing number of sequenced microbial genomes has unveiled a noteworthy prevalence of tellurite-resistant microorganisms. Several tellurite resistance determinants with different gene compositions have thus far been detected (Mittler 2002; Jobling and Ritchie 1987; Whelan et al. 1995; Chen et al. 2004; Ponnusamy and Clinkenbeard 2015; Muñoz-Villagrán et al. 2018; Farias et al. 2022). Surprisingly, a vast number of these microorganisms are pathogenic. One of the tellurite-resistant pathogenic bacteria focused on in our laboratory is *Escherichia coli* KL53. The determinant of this uropathogenic bacterium, consisting of two sets of *ter* genes (*terZABCDEF* and *terWY1XY2Y3*), was utilised for all our experiments (Soltys et al. 2018). Given the absence of any contact with tellurium or its compounds, the reason why gastrointestinal and/or uropathogenic microbes harbour the tellurite resistance gene cluster (TRGC) in their genomes is elusive. However, because of the widespread occurrence among various clades of bacteria, it has been postulated that TRGC mediates an important function not directly related to tellurite resistance.

Reactive oxygen species (ROS) serve as important signal transduction molecules (at low/moderate concentrations) or as toxic by-products of aerobic metabolism (at high concentrations). This concentration is tightly balanced by a vast network of genes, called the ‘ROS gene network’ (Miller et al. 2008). ROS are the most effective antimicrobial components of macrophages to eliminate invading bacteria, so their success in survival is based on their ability to thwart the host’s innate immune response. The uptake of essential metals by cells is crucial for the performance of many metabolic pathways, as well as DNA, RNA, and protein synthesis, which depend on the availability of appropriate metal cofactors. The essential metals in excess cause ROS formation (Hassan and Troxell 2013). High ROS concentrations are extremely harmful to all organisms. Excessive ROS production can cause progressive oxidative damage to all classes of biomacromolecules, such as DNA, all RNA species, membrane lipids, and proteins, which can result in mutagenesis, inhibition of growth, and ultimately cell death (Lloy et al. 1997; Mittler 2002; Fridovich 1978; Seixas et al. 2022). A problem occurs whenever ROS generation and ROS elimination are imbalanced, when the effectiveness of antioxidant defence is insufficient, and the cell is incapable of dealing with the production of reactive oxygen species (Mittler 2002; Sharma et al. 2012). The first antioxidative enzymes occurred during changes in the oxidising environment of Early Earth. As a consequence of their crucial role for all living organisms, they remained evolutionary conserved, which highlights their ancient origin (Cassier-Chauvat et al. 2023).

The key idea of our study was to experimentally show the importance and beneficiary effect of TRGC in surveillance in the oxidative stress environment of pathogenic bacteria. Not only tellurite resistance is triggered by TRGC, but also resistance to bacteriophages, phagocytosis by macrophages, tolerance to oxidative stresses and colicins, adherence to epithelial cells, and the formation of filamentous morphology were also described previously (Whelan et al. 1995; Alonso et al. 2000; Tarr et al. 2000; Yin et al. 2009; Ponnusamy and Clinkenbeard 2015; Peng et al. 2022). This study elucidates the existence of TRGC as a useful tool of the oxidative stress protection mechanism in aerobic bacteria and suggests a possible explanation for this phenomenon. It supports our previous outcomes suggesting benefits for bacteria possessing TRGC in macrophage environment survival (Valkova et al. 2007). Since we sought a deeper understanding of these benefits, we set up experiments to evaluate the range of oxidative damage of TRGC-positive bacterial strains at the protein and lipid levels. To increase the credibility of our hypothesis and the robustness of our results, we determined the expression levels of the ROS scavengers.

## Material and methods

### Bacterial strains and plasmids

**Fig. 1:**
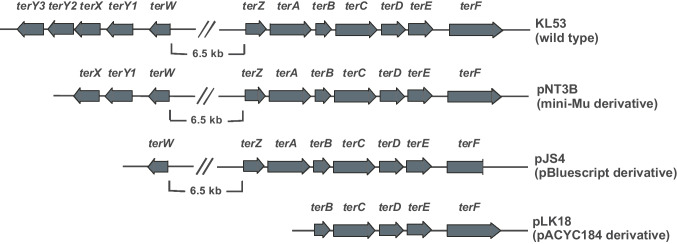
The tellurite resistance gene cluster. The gene composition of *Escherichia coli* KL53 (wild type) plasmid pKL53-L and its derivatives

The bacterial strain *E. coli* KL53 is the original clinical isolate from the Department of Urology, Faculty of Medicine in Bratislava (Burian et al. 1990). This clinical isolate harbours three plasmids (unpublished data). The biggest one, pKL53-L, possesses TRGC, which was used for subcloning. All its derivatives (pLK18, pJS4, and pNT3B) were the results of work published previously (see reference part of Table 1). Gene composition of the clinical wild-type KL53 TRGC and its truncated versions is graphically depicted in Fig. 1. All *E. coli* strains were cultivated in Luria Bertani medium (LB medium) at 37 °C with the addition of the appropriate selection agent. Experimentally used bacterial strains and their gene compositions: laboratory strain MC4100 as the control without TRGC (minimal inhibitory concentration MIC < 0.1 mmol/L (25.37 µg/mL); Valkova et al. 2007); clinical uropathogenic isolate KL53 (GenBank accession no. CP030919.1; MIC 5 mmol/L (1.26 mg/mL); Valkova et al. 2007); derivatives of the largest plasmid pKL53-L (GenBank accession no. CP030920.1) in MC4100, namely, plasmid pNT3B—*terXY1W*, *terZABCDEF* (mini-Mu derivative, MIC—not detected) (Burian et al. 1998), pJS4—*terW*, *terZABCDE*Δ*F* (MIC 5 mmol/L, 1.26 mg/mL) (Vavrova et al. 2006), and pLK18—*terBCDEF* (MIC 2 mmol/L, 0.5 mg/mL) (Burian et al. 1998). The list of *E. coli* strains and plasmids is in Table 1.

**Table 1:** List of *Escherichia coli* bacterial strains and plasmids

**Strain**	**Genotype**	**References**
KL53	Clinical UPEC isolate, *terY3Y2XY1W*, *terZABCDEF*, *terXY1W*, Tc^R^, Sm^R^	Burian et al. (1990)
MC4100	F^−^, *ara*D139, Δ(*arg*F-*lac*) U169, *rps*L150, (Str^R^), *rel*A1, *flb*B5301, *deo*C1, *pts*F25, *rbs*R	Silhavy et al. (1984) purchased from Stratagene

**Plasmids**	**Genotype**	**References**
pNT3B	*terXY1W*, *terZABCDEF*, Tc^R^, Cm^R^, Gm^R^, Te^R^ (mini-Mu derivative)	Burian et al. (1998)
pJS4	*terW*, *terZABCDE*Δ*F* in pBluescript, ColE1, Ap^R^, Te^R^	Vavrova et al. (2006)
pLK18	*terBCDEF* in pACYC184, Cm^R^, Te^R^	Burian et al. (1998)

### Cell viability test

Cell viability was analysed according to the method described by Borghese et al. (2004). The overnight culture was supplemented with K_2_TeO_3_ to a final concentration of 1 mg/mL (3.94 mmol/L) and cultivated for 5 h at 37 °C. The cell suspension was pelleted at 1700 × *g* for 15 min, 4 °C, and the cells were repeatedly washed with 0.9% (w/v) NaCl. The final dilution of the cell suspension was aimed at 0.2 (OD_600_). The bacterial suspension was separately mixed with dyes SYBR Green I (Thermo Fisher Scientific) (diluted 1:100) and propidium iodide (Thermo Fisher Scientific) (final concentration 0.5 mg/mL (0.748 mmol/L)) in a 2:1 ratio and stationarily incubated in the dark for 15 min. The sample was excited at 480 nm, and fluorescence emission was detected at 520 nm for SYBR Green I and 536 nm and 617 nm for propidium iodide. Fluorescence was measured spectrophotometrically using a Tecan Safire II. To correctly evaluate the results, different portions of dead/live cells were prepared by diluting metabolically inactive cells inactivated with 70% (v/v) isopropanol (Stiefel et al. 2015), with live cells washed in 0.9% (w/v) NaCl. Different dilutions of dead/live cells were used for calibration curve establishment.

### Lipid peroxidation assay

The method for determination of lipid peroxidation was based on spectrophotometric quantification of a coloured complex formed in the reaction of malondialdehyde (MDA; Merck) with thiobarbituric acid (TBA; Merck) at 532 nm wavelength. The proteins of crude cell extract were precipitated in 20% (v/v) TCA (1:1). After centrifugation at 12,000 × *g* for 5 min at RT, the protein precipitate was removed, and the supernatant was used in the reaction with TBA reagent (1:1) (saturated TBA in 0.1 mol/L (0.2% (v/v)) HCl and 10 mmol/L (22 mg/mL)) butylated hydroxytoluene (BHT; Merck). The mixtures were heated in a boiling water bath for 1 h. After cooling, the samples were mixed with n-butanol (1:1) and centrifuged at 12,000 × *g* for 5 min at room temperature. The absorbance of the supernatant was measured at 532 nm. TEP was used for standard curve formation (Behuliak et al. 2009; Rice-Evans et al. 1991; Semchyshyn et al. 2005).

### Protein carbonyl content assay

The method used for protein carbonylation measurement was based on protein carbonyl (PCO) derivatization with 2,4-dinitrophenylhydrazine (DNPH). Samples were prepared as crude cell extracts. DNPH solution was prepared by dilution of 10 mmol/L (1.98 mg/mL) DNPH (Merck) in 2.5 mol/L (0.6% (v/v)) HCl. Cell extracts were mixed with DNPH solution (1:4; sample to DNPH solution). As a control, crude cell extracts were mixed only with 2.5 mol/L (0.6% (v/v)) HCl. Samples were incubated in the dark at room temperature for 1 h. Proteins were precipitated with 20% (w/v) trichloroacetic acid (TCA) (1:1), vortexed, and centrifuged (12,000 × *g*, 10 min, room temperature, all centrifugation steps were performed under the same conditions). The pelleted protein samples were repeatedly diluted in 10% (v/v) TCA and after precipitation centrifuged. The last precipitation was performed after dilution in ethanol/ethyl acetate solution (1:1, v/v) and centrifuged. The absorbance (370 nm) of samples diluted in 6 mol/L (573.18 mg/mL) guanidine hydrochloride was measured. The protein carbonyl content was calculated as a difference between the tested and control samples. We used the Beer-Lambert Law and the molar absorption coefficient of DNPH (22,000/M·cm) to quantify the concentration of protein carbonyl groups relative to protein concentration. Carbonyl content was expressed as nanomol carbonyl per milligram of protein (Carty et al. 2000).

### Preparation of the crude cell lysate

Bacterial cultures were grown at 37 °C until reaching the optical density (OD_600_) of approximately 0.45. Cells were harvested by centrifugation (2400 × *g*, 5 min, 4 °C). The cell pellets were washed three times with phosphate buffer solution (PBS; pH 7.2) and resuspended in 1 mL PBS. Cell suspensions were disrupted by sonication (Bandelin Sonopuls HD 3100 homogeniser), and cell debris was removed by centrifugation. Supernatants were used as crude cell extracts (Semchyshyn et al. 2005).

### Protein concentration

Protein concentration was determined by the method described by Bradford (Bradford 1976) using bovine serum albumin (BSA; Thermo Fisher Scientific) as the standard.

### RNA isolation and RT-qPCR

The RNA samples were isolated from the bacterial culture in LB medium without the addition of tellurite (control) and tellurite amended medium 15 min, 45 min, and 90 min after tellurite treatment. The LB medium was supplemented with a sublethal concentration of tellurite (1 mmol/L (253.8 mg/mL) K_2_TeO_3_). Tellurite was added when cells reached the exponential phase 0.6 (OD_600_). Isolated RNA was used as a template for RT qPCR. As endogenous control and for normalisation of the results, two housekeeping genes were chosen: *tufA* (encoding the translation elongation factor EF-Tu 1) from *E. coli* K12 MG1655 (Acc.N.: U00096.2) and *gapA* (encoding glyceraldehyde-3-phosphate dehydrogenase (GAPDH)) from *Escherichia fergusonii* ATCC 35469 (Acc.N.: NC_011740.1). The obtained data were comparable for *tufA* as well as for *gapA* (data for *gapA* are not shown). Primer sequences used in the analyses are summarised in Table 2. The *C*_T_ values were collected with the proprietary software at a constant fluorescence threshold. All RNA samples used were obtained by isolation using the SV Total RNA Isolation kit (Promega) according to the manufacturer’s instructions. The concentration and purity of the RNA were determined using a NanoDrop ND-1000 spectrophotometer (NanoDrop Technologies), and the integrity of RNA was assessed on agarose gel. Isolated RNA was directly used for One-Step qPCR using the QuantiFast SYBR Green RT-PCR kit (Qiagen). All procedures were performed exactly according to the manufacturer’s recommendations, and samples were run in triplicate. The 7900 HT Fast Real-Time PCR System (Applied Biosystems) was used for quantification and SDS 2.2.2 software for data evaluation. The specificity of the qPCR reaction for each amplified product was verified by melting curve analysis. The *C*_T_ values were used to determine the expression level of the target gene in the test sample (tellurite treated) relative to the calibrator sample (in LB medium). According to reaction efficiencies for the reference (housekeeping genes) and target (antioxidative) genes, the 2^−ΔΔCT^ (Livak) method was used for relative gene expression analysis (Livak and Schmittgen 2001).

1. We normalise the C_T_ of the target genes to that of the reference (ref) genes for both the test sample and the calibrator sample:
2. Δ*C*_T(test)_ = *C*_T(target, test)_ − *C*_T(ref, test)_
  Δ*C*_T(calibrator)_ = *C*_T(target, calibrator)_ − *C*_T(ref, calibrator)_
3. Normalise the Δ*C*_T_ of the test sample to the Δ*C*_T_ of the calibrator: ΔΔ*C*_T_ = Δ*C*_T(test)_ − Δ*C*_T(calibrator)_
4. Calculate the expression ratio: 2^−ΔΔC^_T_ = normalised expression ratio

**Table 2:** List of primers and description of selected genes used in real-time qPCR

**Primer**	**Sequence (5′-3′)**	**Protein function**
*gapA_F*	tcg tcc cat ttc agg tta gc	Housekeeping genes
*gapA_R*	cac cgt tga agt gaa aga cg	
*tufA_F*	ccg cag act cgt gag cac at	
*tufA_R*	agc agc tct tcg tca tca acc a	
*fur_F*	cac tga cgt gat ggt tgt cc	Ferric uptake regulator Fur, a transcription factor, which controls expression of ROS damage protective enzymes; important for bacterial virulence
*fur_R*	caa tac cgc cct aaa gaa agc	
*katE_F*	ttt tcc gga ata cga act gg	KatE catalyses the degradation of H_2_O_2_, contributes to H_2_O_2_ resistance
*katE_R*	ttc ttc cgg gat aag ttt gg	
*gorA_F*	ggt atc gcc tcc atc aac c	Gor protein upholds high levels of reduced glutathione in the cytosol
*gorA_R*	cac aca gcc aac att tac gc	
*sodA_F*	tcg gct ccg ttg ata act tc	Encode cytosolic superoxide dismutases which play a major role in protection against oxidative stress
*sodA_R*	gcc agt tta tcg cct ttc ag	

The final result of this method is presented as the fold change of target gene expression in a target sample relative to a reference sample, normalised to a reference gene.

### Data analysis

All data from this study were expressed as means ± standard deviation, and three independent replicates were performed per group. Parametric data were statistically analysed with a Student *t*-test. *P* < 0.05 was set as statistically significant.

## Results

The current study aimed to assess the level of oxidative injury of different *E. coli* strain cells exerted by K_2_TeO_3_ treatment*.* The main idea of our study was experimental confirmation of the beneficial effect of TRGC on pathogenic bacteria in an oxidative stress environment.

### Test of cell viability

The test of cell viability was based on differences in cell membrane integrity of living, dead, or damaged cells and different penetration abilities of the dyes used. Fluorescent dyes SYBR Green I and propidium iodide were used for cell staining. SYBR Green I is a DNA-binding dye that stains cells in all physiological states, whereas propidium iodide can penetrate only the inactive cell membrane of damaged or dead cells (Nebe-von-Caron et al. 2000; Borghese et al. 2004). Potassium tellurite was used for the treatment of tested bacterial strains to trigger oxidative stress conditions. We assume that such conditions mimic the oxidative stress environment within the macrophage (Fang et al. 2016).

**Fig. 2:**
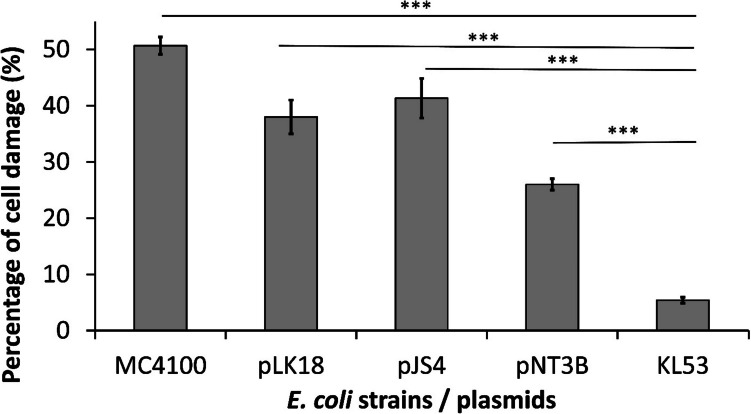
The cell viability test. The viability test of *Escherichia coli* strains MC4100 (tellurite resistance gene cluster negative control), KL53 (clinical uropathogenic isolate, wild type), and *E. coli* MC4100 strains possessing its plasmid pKL53-L derivatives. All experiments were repeated three times and samples were used in triplicate. The average values from the triplicates are shown, with error bars representing the standard deviation. The percentage of damaged cells was compared among *E. coli* MC4100 and other tested strains. Significant differences (*P* < 0.05) were among all compared strains

We tested the membrane integrity of *E. coli* strain KL53 and its subcloned TRGC plasmid derivatives, which were used for transformation of strain MC4100. Laboratory strain MC4100, as the control without TRGC; clinical uropathogenic isolate KL53; derivatives of the largest plasmid pKL53-L (GenBank accession no. CP030920.1) in MC4100, namely, plasmid pNT3B—*terXY1W*, *terZABCDEF* (mini-Mu derivative) (Burian et al. 1998), pJS4—*terW*, *terZABCDE*Δ*F* (Vavrova et al. 2006), and pLK18—*terBCDEF* (Burian et al. 1998), were chosen. The gene composition of the above-mentioned derivatives is depicted in Fig. 1. Tellurite treatment was applied for 5 h, and cell viability was estimated. We detected significant differences in the surveillance of different *E. coli* strains as a result of high-concentration tellurite treatment associated with the *ter* gene genomic content. The control strain, *E. coli* MC4100, exhibited the highest portion of damaged cells (51.26%), whereas the wild clinical strain KL53 had only 6.46% damaged cells. The differences are highly significant among other tested strains, e.g., strain MC4100 pNT3B—*terXY1W*, *terZABCDEF* exhibited 25.69%, MC4100 pLK18—*terBCDEF* 38.05%, and MC4100 pJS4—*terW*, *terZABCDE*Δ*F* 41.17% of viable cells (Fig. 2). To evaluate the results, the calibration curve of different dilutions of metabolically dead/live cells was used. Our study aims to point out the beneficial effects of pathogenic bacteria to be able to survive in ROS-rich environments. The cell viability test clearly showed that bacterial strain KL53 is the most vital in the ROS-rich environment (93.54% of cells can survive) compared with MC4100 laboratory strain (only 48.74% of the cells can survive). All tested plasmids had beneficial effects on cell survival: plasmid pLK18—*terBCDEF* increased survival of strain MC4100 by 13.21%, plasmid pJS4—*terW*, *terZABCDE*Δ*F* 10.09%, plasmid pNT3B—*terXY1W*, *terZABCDEF* 25.57%, respectively. The ability to survive tellurite treatment is the most significant at 44.8% in the wild clinical strain KL53 in comparison with laboratory strain *E. coli* MC4100.

### The measurement of lipid damage—Thiobarbituric acid reactive substance assay

Lipids belong to the group of molecules targeted by free radicals. MDA is a biomarker for enzymatic degradation and lipid peroxidation of polyunsaturated fatty acids. Toxic tellurium compounds cause oxidative injury of cells by increasing cytoplasmic ROS levels. The same *E. coli* strains described above were used to compare the effect of TRGC on lipid peroxidation.

**Fig. 3:**
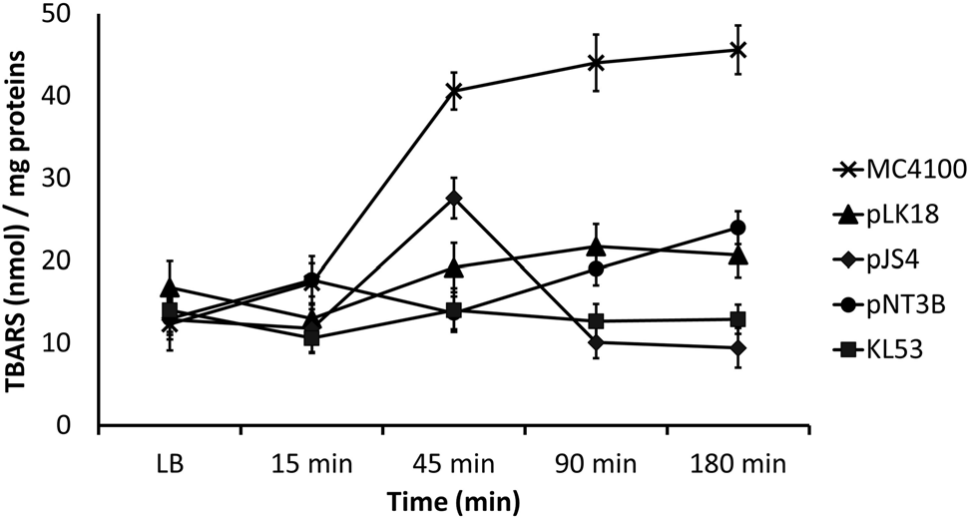
Quantitative determination of thiobarbituric acid reactive substances (TBARS) indicated the oxidative damage of lipids. The *E. coli* strains were supplemented with K_2_TeO_3_, and after 15 min, 45 min, 90 min, and 180 min, lysates were prepared. TBARS concentrations were normalised per milligram of whole-cell proteins estimated using the Bradford reagent assay. The samples were measured as triplicates in three different experiments. Error bars denote the standard deviation of the three replicates

Thiobarbituric acid reactive substance (TBARS) content was measured before and 15, 45, 90, and 180 min after K_2_TeO_3_ treatment. TBARS levels differed due to different genetic profiles. The most evident lipid peroxidation occurred in *E. coli* strain MC4100 (47–48 nmol TBARS/mg protein), which possessed no *ter* gene. The TBARS content increased linearly during the cultivation of this strain in the presence of the toxicant. In contrast, the clinical isolate *E. coli* KL53 with complete (wild type) TRGC exhibited the lowest concentration of TBARS (10–16 nmol TBARS/mg protein). Bacterial strains derived from *E. coli* KL53 exhibited higher lipid damage than the wild-type KL53, which correlated with the complexity of the TRGC. Strain *E. coli* pNT3B—*terXY1W*, *terZABCDEF* demonstrated 19–31 nmol TBARS/mg proteins, *E. coli* pJS4—*terW*, *terZABCDE*Δ*F* 15–21 nmol TBARS/mg proteins, and *E. coli* pLK18—*terBCDEF* 21–39 TBARS/mg proteins after 180 min treatment. The more complete TRGC presented in the bacterial genome, the lower the concentration of TBARS and the lower level of cell lipid peroxidation were detected (Fig. 3). This spectrophotometric quantification of lipid peroxidation was simple and sensitive, however with a low specificity due to the reactivity of MDA and other aldehydes with TBA giving pink-coloured species that absorb at 532 nm (Devasagayam et al. 2003). This is why we supported our results with the second method by Drapper et al. (1993) with the same results (data not shown).

### Protein damage measurement by carbonylation

**Fig. 4:**
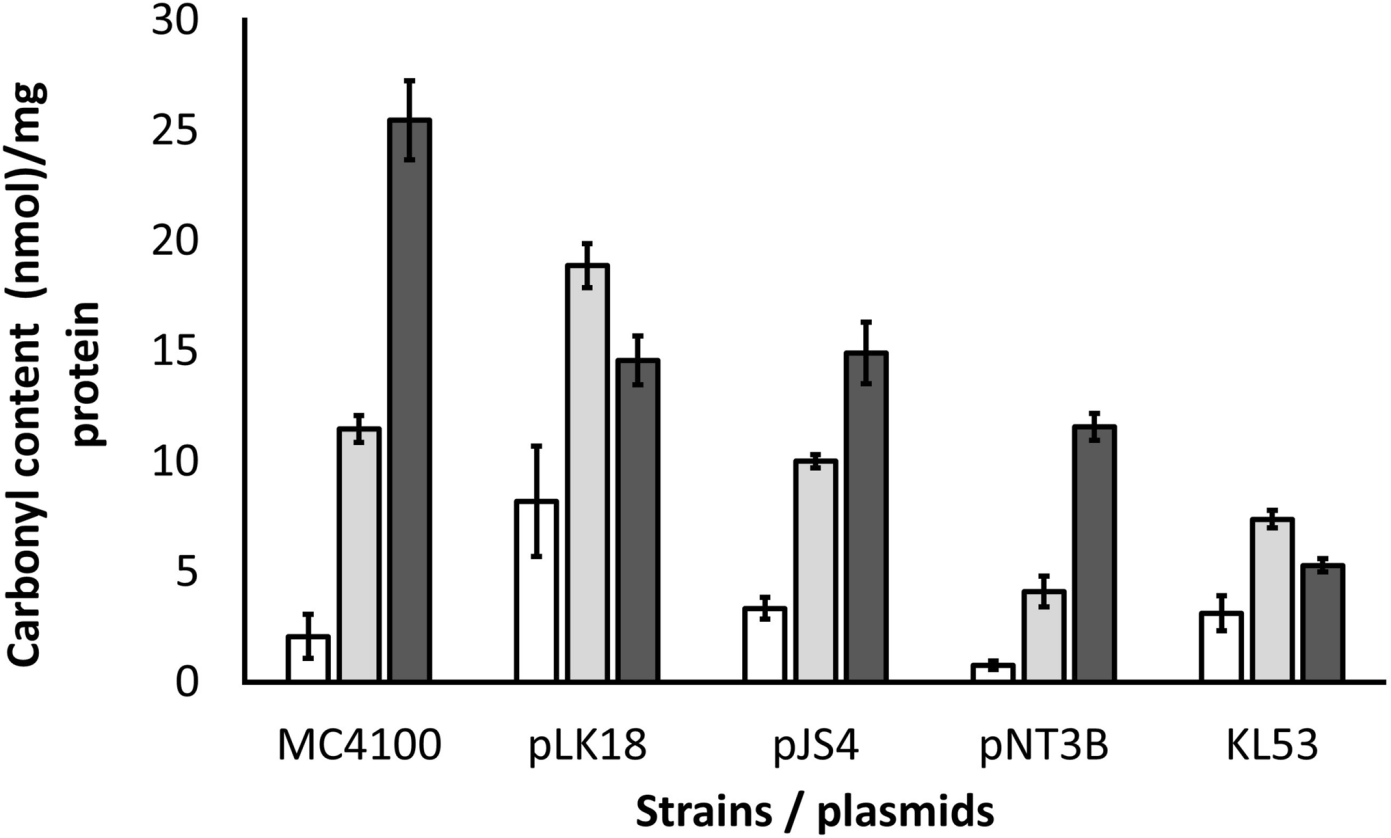
Protein carbonyl content assay. Quantitative determination of protein carbonylation after potassium tellurite treatment of tested bacterial *E. coli* strains. Supplementation of all strains with K_2_TeO_3_ was carried out for 15 min (light grey bars) and 45 min (dark grey bars). Samples before tellurite treatment are shown as white bars. Protein carbonyl content was normalised per milligram of whole-cell protein amount. All experiments were repeated three times and samples were used in triplicate. The average values from triplicates are shown with error bars representing the standard deviation. Significant differences (*P* < 0.05) in carbonyl content were in all tested times (15 and 45 min) except for in LB medium

Irreversible oxidation of proteins due to oxidative stress conditions was used as the basis for carbonylated protein detection. The crude cell extracts of the same *E. coli* strains (control strain MC4100 without any *ter* genes, MC4100 pLK18—*terBCDEF*, MC4100 pJS4—*terW*, *terZABCDE*Δ*F*, MC4100 pNT3B—*terXY1W*, *terZABCDEF*, and *E. coli* KL53) were investigated after cultivation in LB medium with up to 1 mmol/L (253.8 mg/mL) K_2_TeO_3_ added in the exponential phase of growth. We compared average values from triplicate experiments of *E. coli* MC4100 with its transformant after different times of tellurite treatment separately. Significant differences (*P* < 0.05) in carbonyl content were found in all tested times (15 and 45 min), except for the LB medium. The greatest concentration of carbonylated protein (26 nmol/mg protein) was detected after 45 min treatment in control strain *E. coli* MC4100 in comparison with any other tested strains with a different content of *ter* genes (Fig. 4). In *E. coli* strains MC4100 pLK18—*terBCDEF*, MC4100 pJS4—*terW*, *terZABCDE*Δ*F*, and MC4100 pNT3B—*terXY1W*, *terZABCDEF* was detected in values in the range from 13 to 16 nmol/mg protein after 45 min of tellurite treatment. More significant differences were acquired during the earlier phase of measurement, probably due to an increase in the levels of antioxidative enzyme expression and/or reparation mechanisms to be set in. The level of damaged proteins in the KL53 strain is half (65%) that of the MC4100 strain. The duration of tellurite treatment has no significant effect on the trend line of all tested samples.

### Determination of expression levels of selected genes by real-time qPCR

**Fig. 5:**
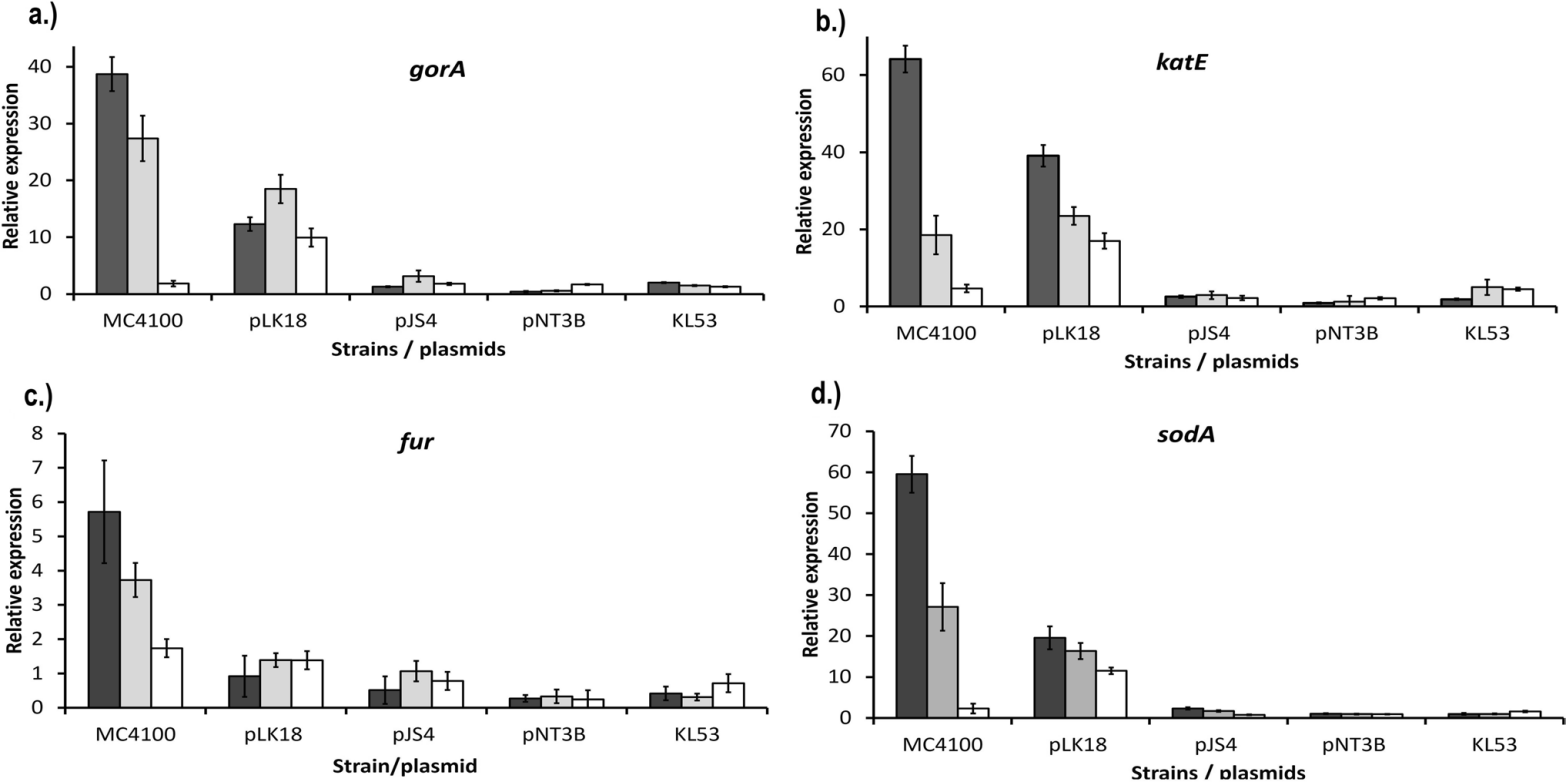
Relative quantification of gene expression. Relative quantification of gene expression of selected antioxidative stress response genes of laboratory strain *E. coli* MC4100 and its transformants harbouring pLK18, pJS4, and pNT3B plasmids and clinical strain KL53 (wild type). The other strains are marked by the names of their plasmids which they harboured. RNA samples were isolated 15 min (dark grey bars), 45 min (light grey bars), and 90 min (white bars) after tellurite exposure. Selected genes encoding enzymes involved in antioxidative cell stress response **a** *gorA*, **b** *katE*, **c** *fur*, and **d** *sodA* were used for relative quantification

To assess the changes in the expression levels of selected antioxidative genes, we used the RT qPCR method. The identical *E. coli* strains as in the previous experiments were tested. We aimed to gain the expression profile of antioxidative genes after tellurite treatment. RNA as templates for One-Step qRT-PCR were isolated for 15 min, 45 min, and 90 min after potassium tellurite addition. The genes encoding enzymes involved in antioxidative stress response—*gorA* (a), *katE* (b), *fur* (c), *sodA* (d)—were used for relative quantification. The genes *tufA* and *gapA* were used for the normalisation of data. The highest expression levels of antioxidative enzymes were measured at the *sodA*, *katE*, and *gorA* genes in control strain MC4100 (without *ter* gene) (Fig. 5). The overexpression of the tested genes reached a 40–70-fold increase compared with housekeeping gene expression. What we did not expect were differences among the tested strains in connection with their TRGC gene portions and different expression levels of the tested antioxidative genes. Their collective participation in the oxidative stress cell response was demonstrated in similar levels of gene expression (up to a 4-fold increase).

Bacterial virulence factors are frequently a part of chromosomes, plasmids, transposons, or temperate bacteriophage DNA and can be integrated into the bacterial chromosome. We consider that *E. coli* KL53 possesses a plasmid pKL53-L with TRGC, which is involved in intricate mechanisms to adapt and survive under various types of abiotic and biotic stress conditions and actively affect the expression of other antioxidative enzymes. The results of all realised analyses lead us to suppose that different parts of TRGC trigger variable levels of oxidative stress gene expression in oxidative stress niches and/or nutrient-deficient niches. All tested antioxidative genes have the same expression pattern depending on the TRGC composition. The highest values of antioxidative gene expression belong to the control strain without *ter* genes. The decreasing values pertain to strains with different *ter* gene compositions due to the reduced portion of *ter* gene determinants. Based on our experimental outcomes, we are persuaded that the genes of TRGC facilitate surviving in a harsh environment that may be poor in nutrients, rich in ROS, etc. These genes participate in eliminating the noxious intracellular H_2_O_2_ and inhibiting intracellular ROS levels that are increasing in the inner space of macrophages. It is possible to consider that ROS generated during tellurite treatment as well as nutrient starvation in the macrophage environment can induce the expression of antioxidative gene pathways.

## Discussion

Our analyses were focused on the measurement of selected parameters that evidence the oxidative damage of the cell due to stress conditions. We also measured the viability of the cells and antioxidative stress enzyme expression levels.

Cellular and membrane integrity is considered a crucial distinguishing feature between dead and viable bacterial cells (Stiefel et al. 2015). Borghese et al. (2004) described the great influence of tellurium compounds on the membrane integrity and plasma membrane redox potential of a cell (Borsetti et al. 2003). The differences in cell membrane integrity of living, dead, or damaged cells and the different penetration abilities of dyes formed the basis for cell viability testing. Our experimental results indicate the beneficial point of the tested plasmids with different truncated versions of TRGC on the viability of the tested strains. It is evident that differences in the TRGC gene composition strongly influence the ability of bacterial strains to survive in an oxidative stress environment and other detrimental effects of ROS generated during tellurite treatment. The bacterial strains harbouring plasmid constructs with more complete TRGC (e.g., pNT3B—*terXY1W*, *terZABCDEF* or pJS4—*terW*, *terZABCDE*Δ*F*) withstand more detrimental effects than the more truncated derivatives (pLK18—*terBCDEF*) of the cluster.

Environmentally generated oxidative stress results in ROS formation which attacks biomacromolecules such as lipids, proteins, and DNA. The oxidative stress normally results in the induction of a variety of antioxidative enzyme expressions in bacteria (Bowler et al. 1992; Mittler et al. 2004; Tremaroli et al. 2007; Zhang et al. 2018). The imbalance between oxidative injury and the ability of antioxidative enzymes at both physiological and DNA levels was measured. The expressions of the set of antioxidative enzymes commonly involved in cell stress response were tested. We determined the expression profiles of different truncated versions of TRGC in the laboratory *E. coli* strain. The most expressed genes were *sodA*, *katE*, and *gorA*, which we expected, since the above-mentioned proteins coded by the tested genes and the metalloproteins SODs play a major role in the protection against oxidative stress. An interesting finding was published by Sun et al. (2016), who found out that *Acinetobacter nosocomialis katE* and *katG* mutant strain is more virulent than the wild-type strain, even if these genes determined H_2_O_2_ resistance; however, catalase is a significant staphylococcal virulence factor (Mandell 1975). Our observation of decreased *katE* expression in a clinical strain mimics the described situation in the *Acinetobacter* strain and leads us to consider the genes of TRGC to be virulent factors. The integral membrane protein TerC protein, a member of TRGC, is considered to be the key protein in tellurite resistance with no significant homology to any protein of known function. An interactome of TerC has been constructed, and TerB was assessed as an interaction partner of TerC. The TerC–TerB complex makes a central unit of functional modules with biochemical activities of C4-dicarboxylate transport, inner membrane stress response, ATPase/chaperone activity, and proteosynthesis (Turkovicova et al. 2016).

Our results also indicate the decreased necessity of *gorA* as well as expression in oxidative stress conditions due to tellurite exposure, the same as in the above-mentioned *fur* and *sodA* genes. More antioxidative genes were tested (*sodB*, *iscS*, *btuE*, *oxyR*, *ompR/envZ*—data not shown) with the same pattern after tellurite treatment but with lower expression levels (from a 12- to 22-fold increase). We pointed out one similarity in viral vs. pathogenic bacteria infection, where we can see a parallel in the effect on the pro-/antioxidant balance in host cells and/or phagocytic cells, respectively, including microbe-induced inhibition of antioxidant enzymes such as superoxide dismutase (Schwarz 1996; Peterhans et al. 1987). Xiao et al. (2012) described *katG* of *E. coli* as a gene that serves for the physiological fitness and pathogenesis-related bacterial survival in macrophages. This finding supports our previous outcomes, which describe the ability of TRGC-positive bacterial strains to survive and propagate in a macrophage environment (Valkova et al. 2007). Several genes, such as *flgE*, *katB*, *agr*, and *ihk-irr*, have been identified as being involved in the intracellular ability to survive of different bacteria (Garzoni and Kelley 2009; Grayfer et al. 2014; Medina et al. 2003; Qin et al. 2014). According to these findings, we analogically suggest that TRGC genes might be genes that heighten physiological fitness, intensify the pathogenic ability to survive, and propagate in host immune cells, which they abuse as shelters to resist drug killing and other immune damage. It is reasonable to suppose that the TRGC genes control ROS levels and that the cells have no reason to heighten the expression of antioxidative defence genes. There is a reason to conclude that the complexity of TRGC has a crucial impact on the lipoperoxidation and protein carbonylation of the tested bacterial strains. According to our outcomes, we come to a general finding, which is likely applicable to more biochemical processes. It follows that the more complete TRGC presented in the bacterial genome, the lower levels of damaged macromolecules, such as proteins or lipids, are carried out.

The presence of tellurite resistance determinants in a wide range of bacterial species suggests that these determinants provide some selective advantage in their natural environment (Walter and Taylor 1992). The function of TRGC and its particular genes has not been elucidated up to date, but it was proved that tellurite resistance determinants provide resistance to bacteriophages and colicins (Whelan et al. 1995; Taylor 1999; Taylor et al. 2002). Vornhagen et al. (2021) used a multidisciplinary approach of genomics and bioinformatics to characterise plasmids harbouring TRGC to determine the biological link between TRGC and *Klebsiella pneumoniae* infection. Their findings indicate that the TRGC encodes factors that resist stress induced by the indigenous human gut microbiota during colonisation. The pathogenic bacteria, e.g., *E. coli* O157:H7, *Yersinia pestis*, *Diphtheria bacillus*, *Deinococcus radiodurans, Staphylococcus aureus*, and *Shigella* spp. (Taylor 1999; Taylor et al. 2002; Valkova et al. 2007; Chasteen et al. 2009; Zhang et al. 2018), incorporated TRGC of different gene compositions into their genomes. These clusters often have features of mobile genetic elements that indicate their mobility potential among genomes via horizontal gene transfer. Moreover, one EHEC *E. coli* O157:H7 strain, EDL933, possesses two identical copies of the genomic island with TRGC (Perna et al. 2001) which has not happened accidentally. TRGC is usually located on a prophage-like element of the chromosome inserted into the tRNA^Ser^, tRNA^Met^, and tRNA^Phe^ positions. They classified TRGC into four subtypes, ter-types 1–4, according to the nucleotide sequence. Only the ter-type 2 operon was located on the IncHI2 plasmid (Nguyen et al. 2021). The resistance to phagocytosis protects bacterial pathogens against host rapid eradication. Since TRGC is considered to be one of the virulence factors, it has been shown that the introduction of TRGC into clinical *E. coli* isolates lacking ter genes improved the fitness of those strains during the macrophage attack (Valkova et al. 2007).

## Conclusion

Based on our experimental results, we suggest an idea for the potential benefit of TRGC in bacterial pathogenicity. We conclude the following:

1. The clinical strain *E. coli* KL53 possesses TRGC, whose gene products might be members of antioxidative enzymes, or they at least allow a decrease or abolishing of the expression of commonly used antioxidative enzymes by unknown mechanisms; in other words, we suggest that all TRGC genes are members of vast ‘ROS gene network’ and contribute to ROS level equilibrium.
2. TRGC promotes bacteria’s ability to survive in stress conditions generated at a different base, which triggers an increase in their fitness in a harsh environment.
3. Oxidative stress conditions do not promote stress signalling in TRGC-harbouring bacteria.
4. TRGC-positive pathogens can affect the host cell or/and phagocytic cell pro-/antioxidant balance by inhibiting the expression of various antioxidant enzymes.
5. The unnecessity or lower necessity of antioxidative enzyme expression depends on TRGC gene completeness.
6. All tested attributes of oxidative damage pointed to a lower level of damage in TRGC-positive bacterial strains.

Therefore, we suggest the protective function of TRGC genes for pathogenic bacteria against a reactive oxygen species-rich phagocytic cell environment. These genes enable the preservation of all tested attributes that tend to be oxidatively injured in an intact undamaged condition or at least allow a lower degree of damage under highly oxidising conditions characterising the respiratory burst of phagocytes.

## Data Availability

The full genome sequences of strains *Escherichia coli* MC4100 (NCBI:txid1403831), KL53 chromosome (NZ_CP030919.1) and its plasmids pKL53-L (NZ_CP030920.1), pKL53-M (NZ_CP030921.1), pKL53-S (NZ_CP030922.1) can be found in Genbank database.
